# Supplementary material for: PGP-14 establishes a polar lipid permeability barrier within the C. elegans pharyngeal cuticle
Source: PLoS Genet. 2023 Nov 6;19(11):e1011008. doi: 10.1371/journal.pgen.1011008 (PMC10653525; doi:10.1371/journal.pgen.1011008)
Supplement: S1 Fig — A. A multiple sequence alignment with the indicated proteins using NCBI’s Clustal Omega tool. All sequences were downloaded from Uniprot and are as follows: Human ABCB1(aka MDR1)(P08183), ABCB4(aka MDR3)(P21439), ABCB5 (Q2M3G0), ABCB11 (BSEP)(O95342), and C. elegans PGP-2 (G5EDF9), PGP-9 (G5EG58), PGP-12 (Q19733), PGP-13 (Q19734), PGP-14 (G5EG61). Black underline indicates the alpha-helixes that are the presumptive PGP-14 transmembrane domains as predicted by Alpha-fold. Red underline indicates the ABC nucleotide binding domain as predicted by the SMART protein database. Also highlighted are the non-sense (red highlight) and missense (blue highlight) mutations identified in the forward genetic screen. The blue boxes around the select residues of ABCB4 are those that interact with phosphatidylcholine as shown by cryo-EM [64]. The blue and green boxes around the select residues of ABCB11 are those that interact with bile acid as shown by cryo-EM [79]. B. Multiple sequence alignments of select regions of sequence of all C. elegans 14 PGPs aligned with each of three human ABCBs. The alignments show only the residues that have been implicated in substrate binding of the respective ABCB transporter. The ABCB1 (MDR1) alignment shows the residues (black boxes) important for interaction with taxol [32] and/or vincristine [33]. The ABCB4 alignment shows residues (black boxes) that interact with phosphatidylcholine [64]. The ABCB11 (BSEP) alignment shows residues (black boxes) important for interaction with bile salt [79]. The Uniprot entries used are as follows: PGP-1 (P34712), PGP-2 (G5EDF9), PGP-3 (P34713), PGP-4 (Q20331), PGP-5 (Q17645), PGP-6 (Q22656), PGP-7 (Q22655), PGP-8 (G5EG03), PGP-9 (G5EG58), PGP-10 (Q18824), PGP-11 (Q19015), PGP-12 (Q19733), PGP-13 (Q19734), PGP-14 (G5EG61), ABCB1(aka MDR1)(P08183), ABCB4(aka MDR3)(P21439), ABCB5 (Q2M3G0), and ABCB11 (BSEP)(O95342). In the alignment, red letters represent small and hydrophobic residues, blue letters represent acid [file pgen.1011008.s001.docx]

**
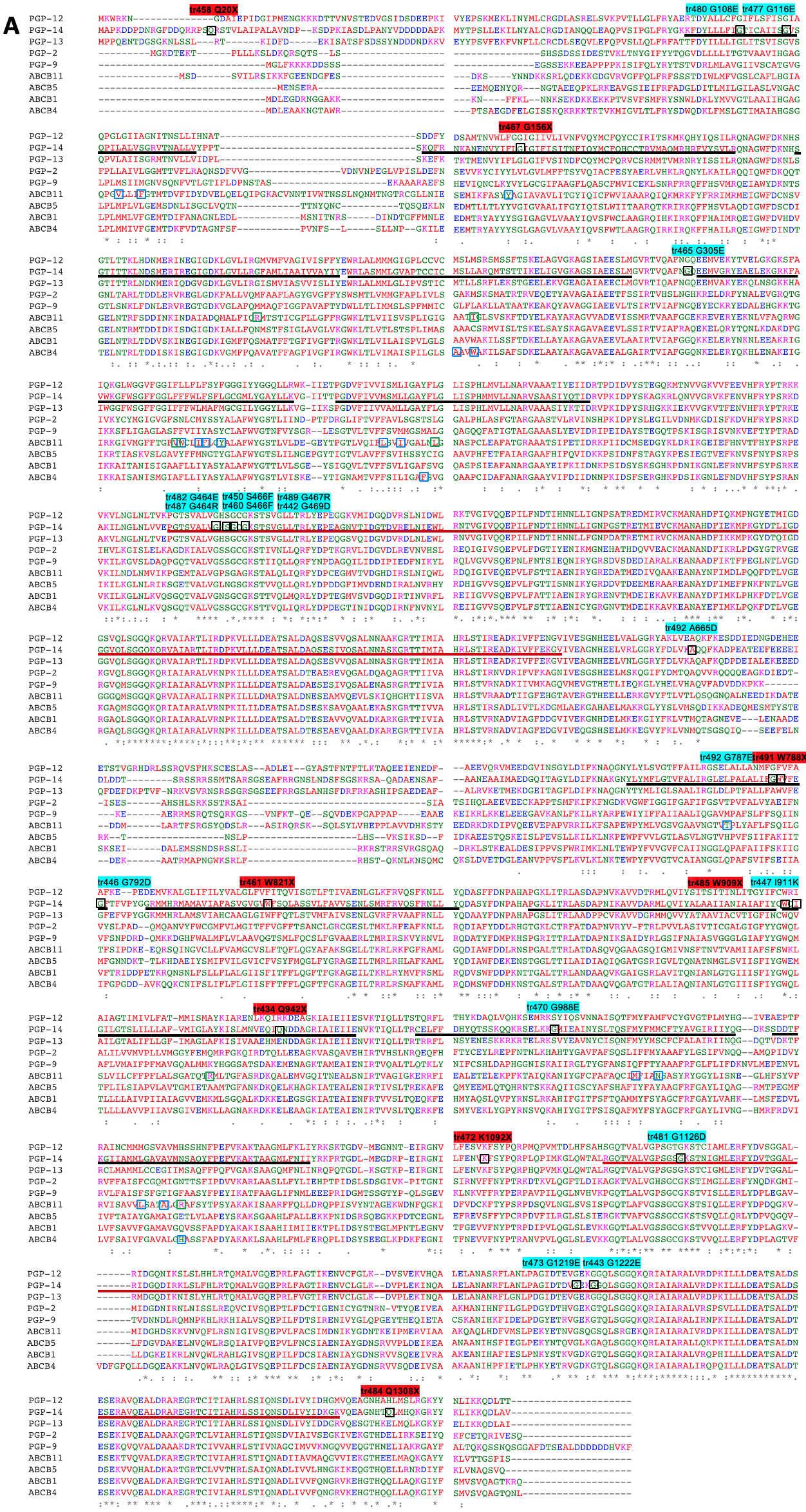
**

**
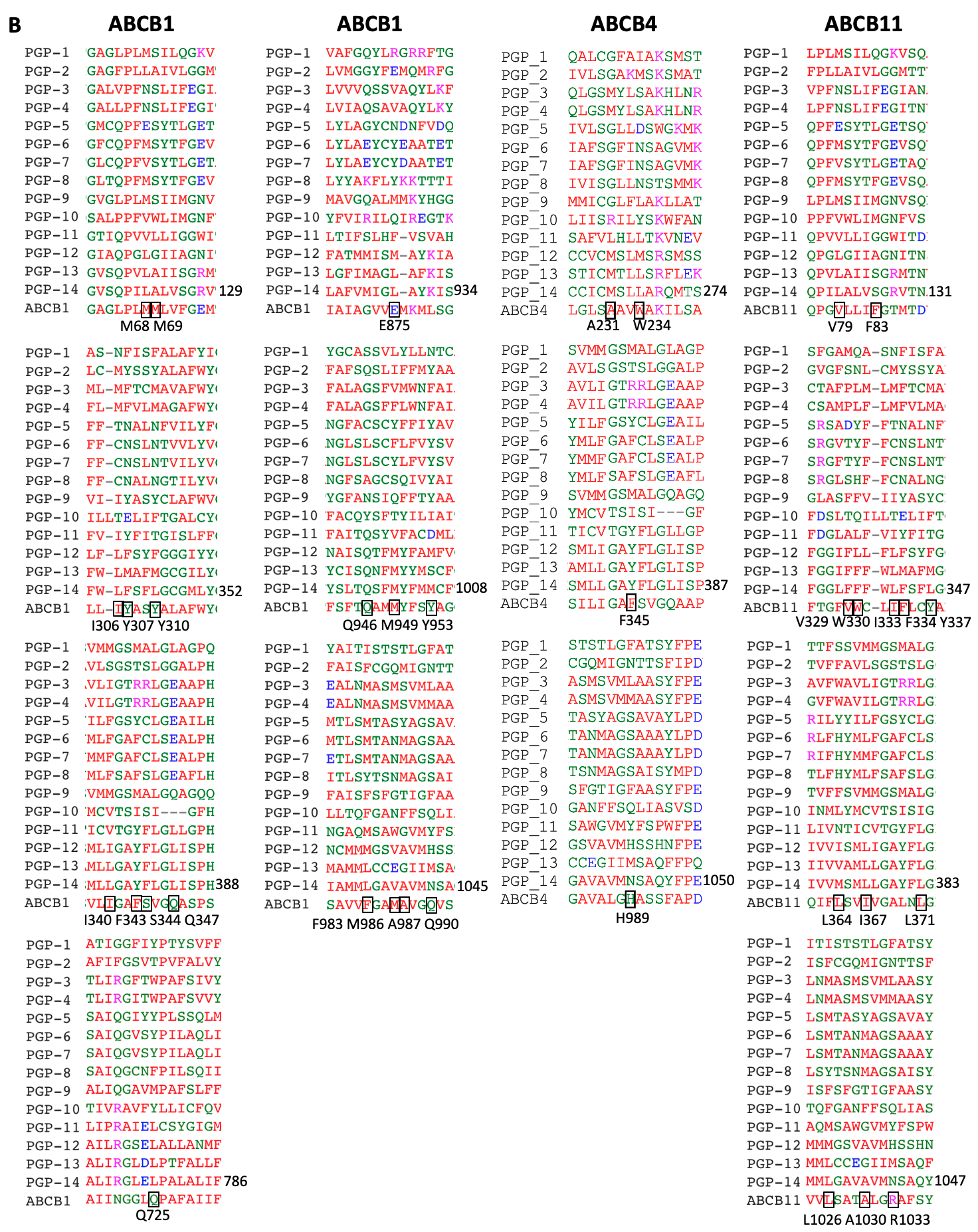
**

**
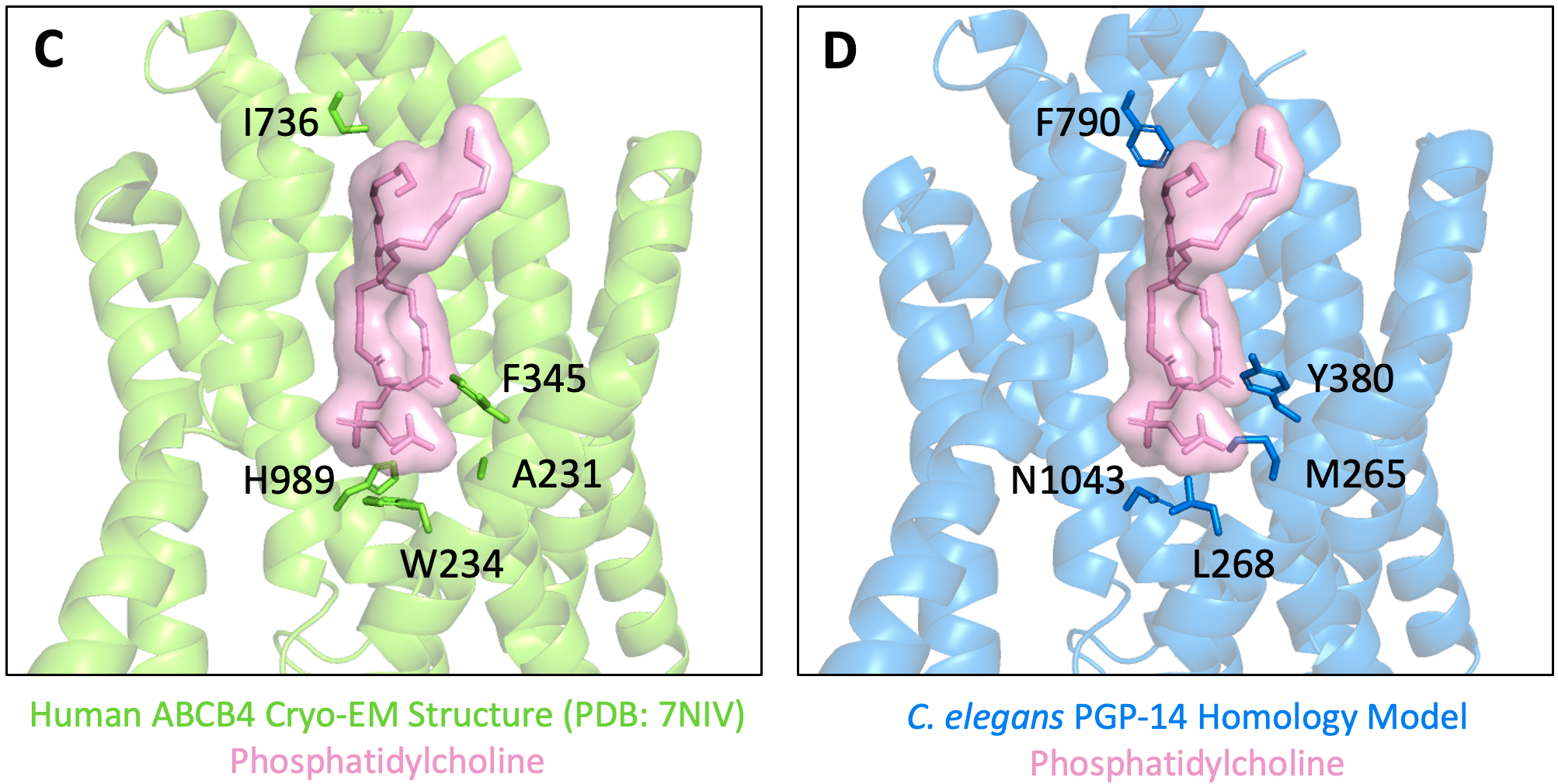
**

**S1 Fig. Multiple Sequence Alignments with PGP-14. A.** A multiple sequence alignment with the indicated proteins using NCBI’s Clustal Omega tool. All sequences were downloaded from Uniprot and are as follows: Human ABCB1(aka MDR1)(P08183), ABCB4(aka MDR3)(P21439), ABCB5 (Q2M3G0), ABCB11 (BSEP)(O95342), and *C. elegans* PGP-2 (G5EDF9), PGP-9 (G5EG58), PGP-12 (Q19733), PGP-13 (Q19734), PGP-14 (G5EG61). Black underline indicates the alpha-helixes that are the presumptive PGP-14 transmembrane domains as predicted by Alpha-fold. Red underline indicates the ABC nucleotide binding domain as predicted by the SMART protein database. Also highlighted are the non-sense (red highlight) and missense (blue highlight) mutations identified in the forward genetic screen. The blue boxes around the select residues of ABCB4 are those that interact with phosphatidylcholine as shown by cryo-EM [64]. The blue and green boxes around the select residues of ABCB11 are those that interact with bile acid as shown by cryo-EM [79]. **B.** Multiple sequence alignments of select regions of sequence of all *C. elegans* 14 PGPs aligned with each of three human ABCBs. The alignments show only the residues that have been implicated in substrate binding of the respective ABCB transporter. The ABCB1 (MDR1) alignment shows the residues (black boxes) important for interaction with taxol [32] and/or vincristine [33]. The ABCB4 alignment shows residues (black boxes) that interact with phosphatidylcholine [64]. The ABCB11 (BSEP) alignment shows residues (black boxes) important for interaction with bile salt [79]. The Uniprot entries used are as follows: PGP-1 (P34712), PGP-2 (G5EDF9), PGP-3 (P34713), PGP-4 (Q20331), PGP-5 (Q17645), PGP-6 (Q22656), PGP-7 (Q22655), PGP-8 (G5EG03), PGP-9 (G5EG58), PGP-10 (Q18824), PGP-11 (Q19015), PGP-12 (Q19733), PGP-13 (Q19734), PGP-14 (G5EG61), ABCB1(aka MDR1)(P08183), ABCB4(aka MDR3)(P21439), ABCB5 (Q2M3G0), and ABCB11 (BSEP)(O95342). In the alignment, red letters represent small and hydrophobic residues, blue letters represent acidic residues, magenta letters indicate basic residues, and green letters represent hydroxyl, sulfhydryl and amine residues. In each alignment, the last residue of PGP-14 is indicated to the right for reference. **C.** Cryo-EM structure of human ABCB4 (PDB ID 7NIV) [64] phosphatidylcholine (PC) binding pocket. ABCB4 is shown as ribbons (green), with key residues of the binding pocket labeled and shown as sticks. Bound PC is shown as sticks with an overlaid surface representation (pink). **D.** A *C. elegans* PGP-14 homology model of the ABCB4 PC binding pocket. This PGP-14 homology model is overlaid with the bound PC structure shown in (C). PGP-14 is shown as ribbons (blue). The PGP-14 residues aligning with those highlighted in (C) are labeled and shown as sticks. Key residues surrounding the choline moiety in the ABCB4 binding site (A231, W234, F345, H989) do not share identity between ABCB4 and PGP-14. This may impact recognition of PC and/or result in steric hindrance to PC binding. Further steric clashes in the binding site may occur as the result of bulkier side chains in PGP-14 than the corresponding ABCB4 residue (ex. I736/F790).
