# Supplementary material for: PGP-14 establishes a polar lipid permeability barrier within the C. elegans pharyngeal cuticle
Source: PLoS Genet. 2023 Nov 6;19(11):e1011008. doi: 10.1371/journal.pgen.1011008 (PMC10653525; doi:10.1371/journal.pgen.1011008)
Supplement: S2 Fig — A phylogenetic tree of the indicated proteins constructed and displayed using MEGA11 [80] and ITOL [81] software. C. elegans proteins are highlighted in blue; human ABCB family proteins are highlighted in red. The pairwise blastp percentage identity (using NCBI protein blast tool) between PGP-14 and the four human ABCB family members is indicated next to the respective human protein names. The Uniprot entries used to create the phylogenetic tree and to make the pairwise blastp comparisons are the same described in S1 Fig. (PDF) [file pgen.1011008.s002.pdf]

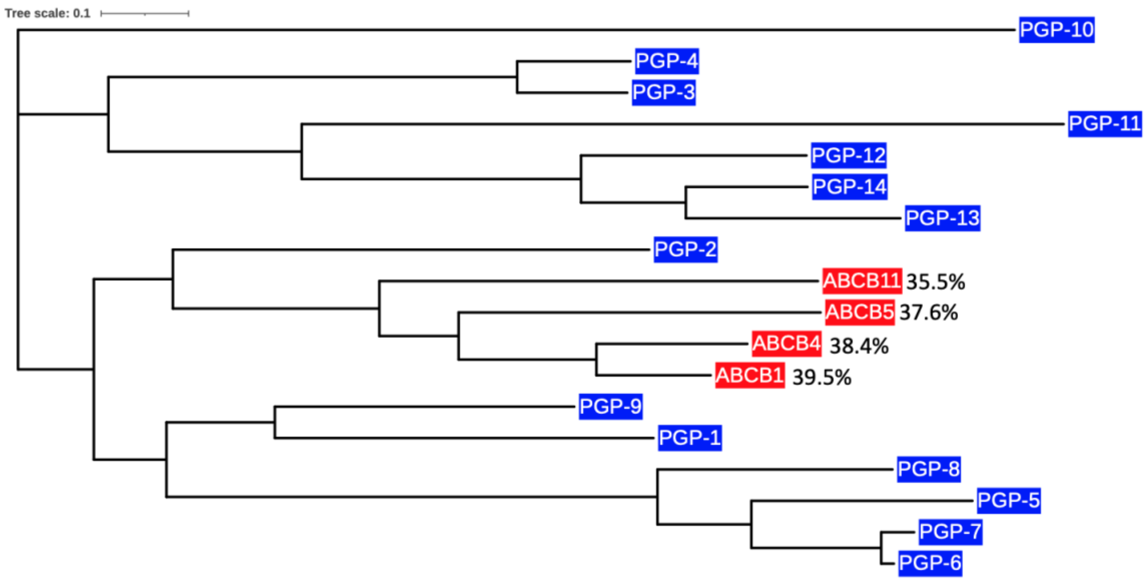

S2 Fig. A Phylogenetic Tree Analysis of *C. elegans* and Human ABCB Family Members. A phylogenetic tree of the indicated proteins constructed and displayed using MEGA11 [80] and ITOL [81] software. *C. elegans* proteins are highlighted in blue; human ABCB family proteins are highlighted in red. The pairwise blastp percentage identity (using NCBI protein blast tool) between PGP-14 and the four human ABCB family members is indicated next to the respective human protein names. The Uniprot entries used to create the phylogenetic tree and to make the pairwise blastp comparisons are the same described in S1 Fig.
