# Supplementary material for: PGP-14 establishes a polar lipid permeability barrier within the C. elegans pharyngeal cuticle
Source: PLoS Genet. 2023 Nov 6;19(11):e1011008. doi: 10.1371/journal.pgen.1011008 (PMC10653525; doi:10.1371/journal.pgen.1011008)
Supplement: S3 Fig — Top and middle sections: The behavior of 25 mutants harboring mutant genes of the ABC family, including 13 other pgp genes and the pgp-15 pseudogene on solid or liquid media containing the indicated concentration of wact-190. Bottom section: Nine other missense mutations that were randomly generated in the million mutation project [28] that do not resist the lethality of wact-190, suggesting that the mutations fail to create a hypomorphic state. (PDF) [file pgen.1011008.s003.pdf]

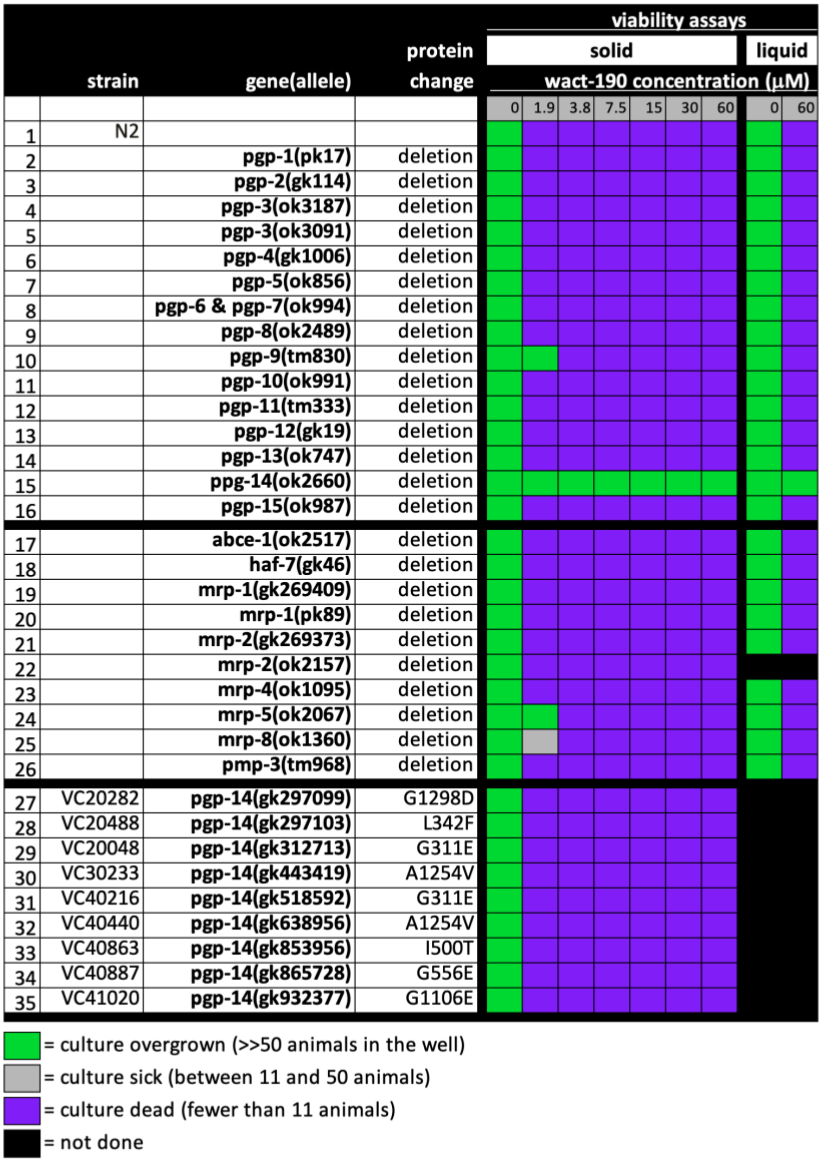

Supplementary Figure 3. Wact-190-Resistance is Specific to *pgp-14* Hypomorphic Mutations. Top and middle sections: The behavior of 25 mutants harboring mutant genes of the ABC family, including 13 other *pgp* genes and the *pgp-15* pseudogene on solid or liquid media containing the indicated concentration of wact-190. Bottom section: Nine other missense mutations that were randomly generated in the million mutation project [28] that do not resist the lethality of wact-190, suggesting that the mutations fail to create a hypomorphic state.
