# Supplementary material for: PGP-14 establishes a polar lipid permeability barrier within the C. elegans pharyngeal cuticle
Source: PLoS Genet. 2023 Nov 6;19(11):e1011008. doi: 10.1371/journal.pgen.1011008 (PMC10653525; doi:10.1371/journal.pgen.1011008)
Supplement: S4 Fig — An RP3510 trEx1010[pPRHZ1138(pgp-14p::SMS-5::FLAG::mCherry); pPRHZ1144(pgp-14p::YFP-PGP-14)] adult worm stained with calcofluor white (CFW) highlighting that PGP-14 is expressed exclusively in the anterior pharynx. A. DIC image. B. The channel showing tagged-PGP-14 expression. C. The overlap in signal between tagged-PGP-14 and CFW. Orange arrows indicate the buccal cavity, red arrows indicate tagged-PGP-14 expression, and the yellow arrows highlight the pharynx terminal bulb grinder. (PDF) [file pgen.1011008.s004.pdf]

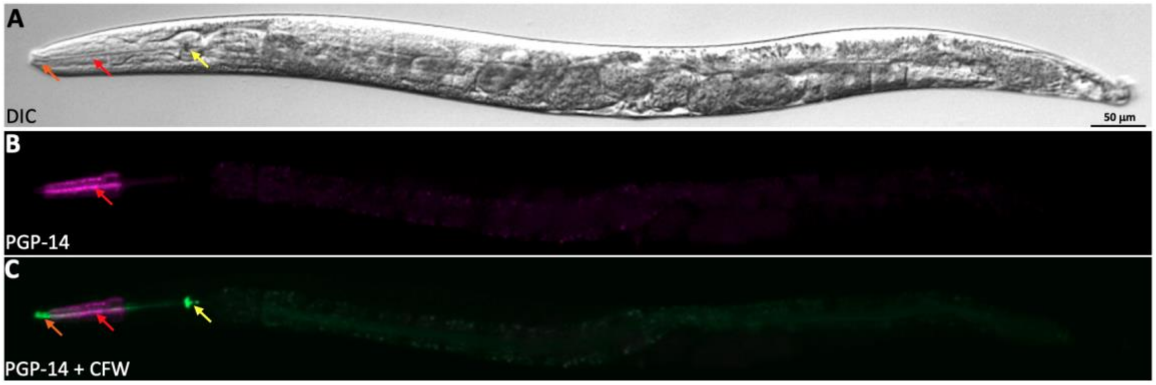

S4 Fig. PGP-14 Expression is Restricted to the Anterior Pharynx. An RP3510 *trEx1010*[pPRHZ1138(*pgp-14p*::SMS-5::FLAG::mCherry); pPRHZ1144(*pgp-14p*::YFP-PGP-14)] adult worm stained with calcofluor white (CFW) highlighting that PGP-14 is expressed exclusively in the anterior pharynx. **A.** DIC image. **B.** The channel showing tagged-PGP-14 expression. **C.** The overlap in signal between tagged-PGP-14 and CFW. Orange arrows indicate the buccal cavity, red arrows indicate tagged-PGP-14 expression, and the yellow arrows highlight the pharynx terminal bulb grinder.
