# Supplementary material for: PGP-14 establishes a polar lipid permeability barrier within the C. elegans pharyngeal cuticle
Source: PLoS Genet. 2023 Nov 6;19(11):e1011008. doi: 10.1371/journal.pgen.1011008 (PMC10653525; doi:10.1371/journal.pgen.1011008)
Supplement: S5 Fig — A-F. The head of an RP3510 trEx1010[pPRHZ1138(pgp-14p::SMS-5::FLAG::mCherry); pPRHZ1144(pgp-14p::YFP-PGP-14)] animal before the L4 to adult molt (45 hours after synchronization at 20°C). G-L. The head of an RP3510 just after the L4 to adult molt (the animal has one embryo in its uterus) (54 hours after synchronization). DIC, differential interference contrast; calcofluor white (CFW) and the functional tagged SMS-5 are shown in green; functional tagged PGP-14 is shown in fuchsia. Merge 1 is the merge of the PGP-14 and SMS-5 micrographs, and merge 2 is the merge of the PGP-14 and CFW micrographs. In all panels, orange arrows indicate the buccal cavity; the red arrows indicate an apical plane of the channels and marginal cells; the pink arrows indicate a basal lane of the marginal cells; the white arrows indicate the signal overlap between PGP-14 and SMS-5 that can be seen at the presumptive plasma membrane. The brightness and contrast of CFW before (B) and after (H) the molt are not manipulated (hence the 1X notation); the brightness and contrast of the tagged-PGP-14 signal before the molt (C) had to be increased by 50% to be clearly visible on the figure (hence the 1.5X notation); the brightness and contrast of the tagged-PGP-14 signal shortly after the molt (I) was not manipulated (hence the 1X notation); the lowly-expressed tagged-SMS-5 signal has been digitally increased to allow visibility (hence the >2X notation). M. Quantification of YFP::PGP-14 expression in animals occurred over two trials with the indicated number of animals in each trial (each dot represents a single animal) during the inter-molt period and with animals that had clear evidence of molting (molt) (see methods for details). A Student’s t-test was used to measure the significance of the difference. (PDF) [file pgen.1011008.s005.pdf]

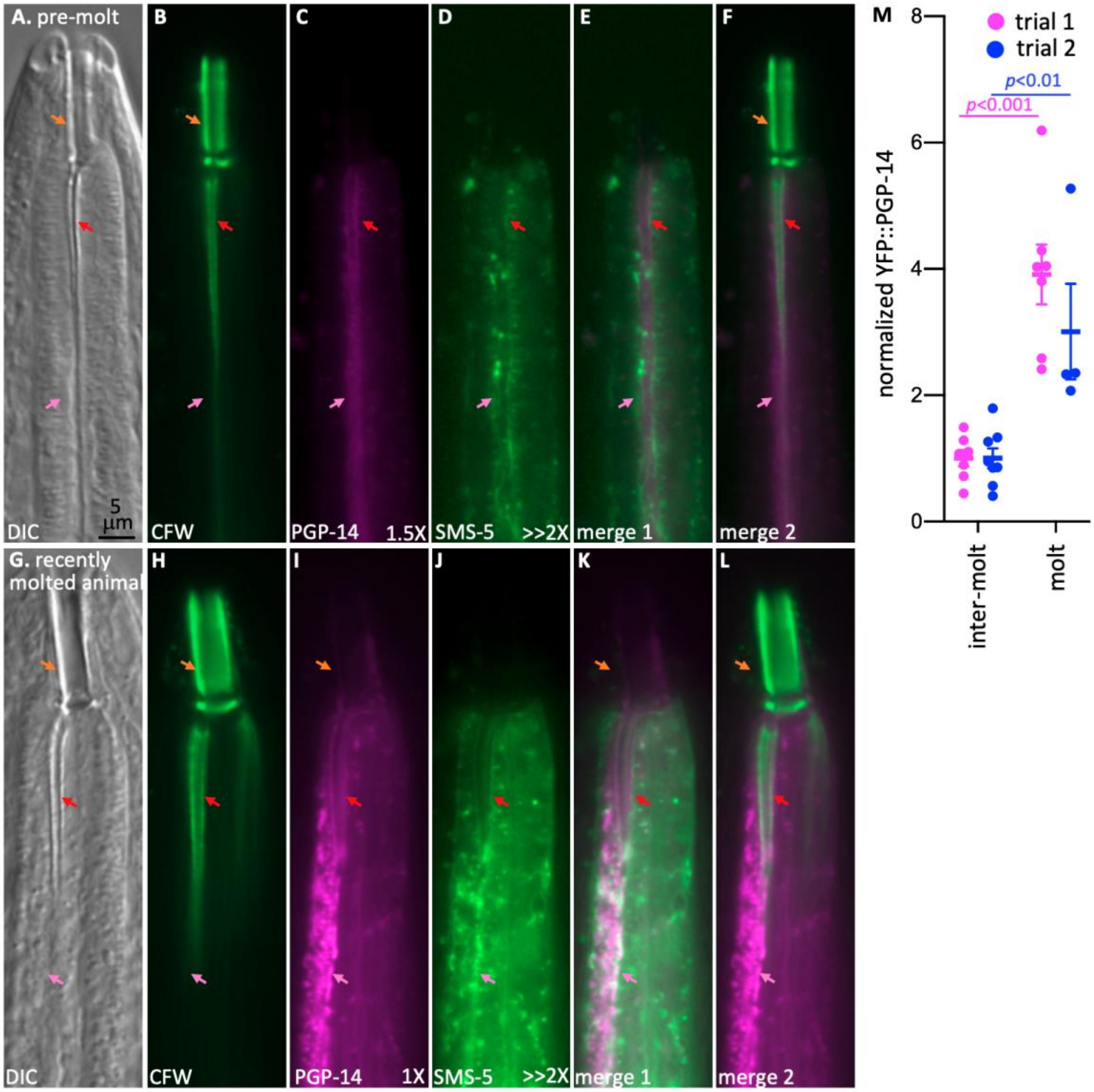

S5 Fig. Additional Examples of the Localization of tagged-PGP-14 and SMS-5. A-F. The head of an RP3510 *trEx1010*[pPRHZ1138(*pgp-14p*::SMS-5::FLAG::mCherry); pPRHZ1144(*pgp-14p*::YFP-PGP-14)] animal before the L4 to adult molt (45 hours after synchronization at 20°C). **G-L.** The head of an RP3510 just after the L4 to adult molt (the animal has one embryo in its uterus) (54 hours after synchronization). DIC, differential interference contrast; calcofluor white (CFW) and the functional

tagged SMS-5 are shown in green; functional tagged PGP-14 is shown in fuchsia. Merge 1 is the merge of the PGP-14 and SMS-5 micrographs, and merge 2 is the merge of the PGP-14 and CFW micrographs. In all panels, orange arrows indicate the buccal cavity; the red arrows indicate an apical plane of the channels and marginal cells; the pink arrows indicate a basal lane of the marginal cells; the white arrows indicate the signal overlap between PGP-14 and SMS-5 that can be seen at the presumptive plasma membrane. The brightness and contrast of CFW before (B) and after (H) the molt are not manipulated (hence the 1X notation); the brightness and contrast of the tagged-PGP-14 signal before the molt (C) had to be increased by 50% to be clearly visible on the figure (hence the 1.5X notation); the brightness and contrast of the tagged-PGP-14 signal shortly after the molt (I) was not manipulated (hence the 1X notation); the lowly-expressed tagged-SMS-5 signal has been digitally increased to allow visibility (hence the >2X notation). **M.** Quantification of YFP::PGP-14 expression in animals occurred over two trials with the indicated number of animals in each trial (each dot represents a single animal) during the inter-molt period and with animals that had clear evidence of molting (molt) (see methods for details). A Student's t-test was used to measure the significance of the difference.
