# Supplementary material for: PGP-14 establishes a polar lipid permeability barrier within the C. elegans pharyngeal cuticle
Source: PLoS Genet. 2023 Nov 6;19(11):e1011008. doi: 10.1371/journal.pgen.1011008 (PMC10653525; doi:10.1371/journal.pgen.1011008)
Supplement: S1 Data — The missense mutations are shown in columns L through AJ. The deletion alleles and rearrangements are shown in columns AL-AO. The data are summarized in Fig 3A in the main display items. Tab B. The 2716 C. elegans Genes that Oscillate in their Expression. The 2716 genes that were previously described to oscillate in their expression in a regular pattern at each larval stage [39] are shown. Column H shows the relative time at which the gene peaks in expression and column G shows the relative amplitude of the expression level. Genes whose expression is enriched during the L4-to-adult molt [15] are indicated in column J. Genes whose expression is enriched in the pharynx [16, 35] are indicated in column K. Genes that encode a signal peptide are shown column L. The data are summarized in Fig 5 in the main display items. Tab C. The 508 Chembridge Inc Small Molecules Surveyed for Sensitivity in wild type and pgp-14(ok2660) and sms-5(ok2498) Null Mutants. Each strain was tested against each molecule four times in 96 well plate format. Each well is seeded with 20 synchronized L1 animals and scored 6 days later. A score of 9 (green) indicates the culture was overgrown (>>50 animals). A score of 0 indicates the culture had 11 or fewer animals (purple). The number of animals in wells with between 11 and 50 are indicated. The class of molecule (resistant, hypersensitive, etc) is shown in column E. These data are summarized in Fig 7E in the main display items. Tab D. Predictions of whether the 48 Chembridge Inc Small Molecules (Wactives) that Form Crystals are Likely MDR1 (aka PGP, aka ABCB1) Substrates. The 48 crystalizing wactives and their Chembridge identification numbers and SMILES structure are shown in columns A-D. The SwissADME [34] predictions for each molecule are shown in column M. Tab E. The Predicted Hydrophobicity of 38 Crystalizing Molecules Compared to the 188 Hypersensitive Molecules. The predicted hydrophobicity of the crystalizing and hypersensitive molecules i [file pgen.1011008.s006.docx]

**S1 Data**

**Tab 1. The Whole-Genome Sequencing Data of *pgp-14* Mutants that were Recovered in Screens for Mutants that Resist wact-190.** The missense mutations are shown in columns L through AJ. The deletion alleles and rearrangements are shown in columns AL-AO. The data are summarized in Figure 3a in the main display items.

**Tab 2. The 2716 *C. elegans* Genes that Oscillate in their Expression.** The 2716 genes that were previously described to oscillate in their expression in a regular pattern at each larval stage [39] are shown. Column H shows the relative time at which the gene peaks in expression and column G shows the relative amplitude of the expression level. Genes whose expression is enriched during the L4-to-adult molt [15] are indicated in column J. Genes whose expression is enriched in the pharynx [16, 35] are indicated in column K. Genes that encode a signal peptide are shown column L. The data are summarized in Figure 5 in the main display items.

**Tab 3. The 508 Chembridge Inc Small Molecules Surveyed for Sensitivity in wild type and *pgp-14(ok2660)* and *sms-5(ok2498)* Null** **Mutants.** Each strain was tested against each molecule four times in 96 well plate format. Each well is seeded with 20 synchronized L1 animals and scored 6 days later. A score of 9 (green) indicates the culture was overgrown (>>50 animals)**.** A score of 0 indicates the culture had 11 or fewer animals (purple). The number of animals in wells with between 11 and 50 are indicated. The class of molecule (resistant, hypersensitive, etc) is shown in column E. These data are summarized in Figure 7e in the main display items.

**Tab 4. Predictions of whether the 48 Chembridge Inc Small Molecules (Wactives) that Form Crystals are Likely MDR1 (aka PGP, aka ABCB1) Substrates.** The 48 crystalizing wactives and their Chembridge identification numbers and SMILES structure are shown in columns A-D. The SwissADME [34] predictions for each molecule are shown in column M.
