# Supplementary material for: PGP-14 establishes a polar lipid permeability barrier within the C. elegans pharyngeal cuticle
Source: PLoS Genet. 2023 Nov 6;19(11):e1011008. doi: 10.1371/journal.pgen.1011008 (PMC10653525; doi:10.1371/journal.pgen.1011008)
Supplement: Attachment — Submitted filename: PGP-14-Response to the Reviews-final.pdf [file pgen.1011008.s007.pdf]

We have taken the liberty of changing the order of the Reviewers for our responses. The original reviewer numbers remain the same.

Reviewer #2-Opening Remarks: Most exposed epithelial surfaces are coated with an apical extracellular matrix (aECM) that confers barrier functions to protect against xenobiotics. This aECM barrier typically contains lipid components, though in many cases it is poorly understood what those lipids are, where they are located in the matrix, or how they get there. For example, in *C. elegans*, there are several different types of aECM that have barrier functions (eggshell, body cuticle, pharyngeal cuticle, intestinal glycocalyx), but none of these have well-defined lipid content or mechanisms of barrier assembly.

Here Kamal et al provide convincing evidence that the ABCB4-type transporter PGP-14 is needed to establish a proper xenobiotic barrier in the *C. elegans* pharynx cuticle, and that it acts in a common process with the sphingomyelin synthase SMS-5. Both proteins are expressed specifically in the pharynx, with peak expression during cuticle synthesis, and loss-of-function mutants have heightened sensitivity to a large number of xenobiotics, but reduced sensitivity to a smaller set of crystal-forming toxins. PGP-14 is related to a well-characterized phospholipid transporter, and reduced phospholipid content of the pharynx cuticle in mutants is inferred based on reduced staining with Nile Red and with a sphingomyelin sensor. Overall, the data appear rigorous and represent a significant step forward in understanding the biology of the pharyngeal cuticle - it will be of broad interest to cell and matrix biologists, especially those interested in lipid transporters. I am quite enthusiastic and have only minor suggestions to improve the manuscript.
Our Response: We thank the reviewer for the insightful summary and kind words.

Reviewer #2-Comment 1: There isn't any direct evidence for a specific affinity of PGP-14 for PC or for a change in PC levels or distribution in the mutants. Therefore I recommend toning down the suggestion that PC is the relevant phospholipid.
Our Response: We do not disagree with the Reviewer on this point. We were careful in the original submission to avoid any claim that phosphatidylcholine (PC) is the lipid being transported by PGP-14. The strongest statements to this effect in the original manuscript has to do with the GFP::NT-lysenin experiment, both in the Results and Discussion sections. We have made an effort to add additional cautiousness in both of the relevant sections, and report those changes here:

Revised paragraph in the Results section (changes are underlined):

> *Given the homology between PGP-14 and ABCB4, we asked whether PGP-14 might flop PCs in vivo. We previously demonstrated that sms-5(0) has a deficit of clustered sphingomyelin (SM) on the pharynx (Kamal et al., 2019) using a protein probe called GFP::NT-lysenin (Ishitsuka et al., 2004; Yilmaz et al., 2018) <u>on animals whose cells were not permeabilized</u>. The active site of the SM synthases resides on the outer leaflet of membranes where it uses PC as a substrate to make SM (Tafesse et al., 2006). If PGP-14 flop PCs to the outer leaflet, then pgp-14(0) might also have a deficit in SM abundance <u>as detected by a decreased GFP::NT-lysenin signal</u>. Indeed, we observe a significant decrease in GFP::NT-lysenin signal in the pgp-14(0) mutant background (p=0.004; Fig 6I-6J). The fold decrease in clustered SM in pgp-14(0) is not as dramatic as that observed in the sms-5 null background (Kamal et al., 2019). These observations suggest that SMS-5 uses other substrates beyond what PGP-14 may provide, which is not unexpected. Regardless, this data is consistent with the <u>hypothesis</u> that PGP-14 <u>may</u> flop PC in vivo.*

Revised paragraph in the Discussion section (changes are underlined):

While our NT-lysenin results suggest that PGP-14 may flop PCs to the outer leaflet along the pharynx plasma membrane (see Figure 6J), it is entirely possible that PGP-14 exports other phospholipids, which may or may not include PCs, to the developing pharynx cuticle.

We hope these changes are satisfactory.

Reviewer #2-Comment 2: The MS starts out with a description of small molecule-induced toxic crystal formation and the isolation of *pgp-14* mutants in a genetic screen for resistance to this. It's a beautiful example of the often unexpected insights that come from forward genetics, but it's also a rather circuitous route to the topic of aECM lipid content. It wasn't until very late in the MS (e.g. Discussion lines 366-371) that the authors clearly explained the likely connection between these topics (that hydrophobic lipids repel hydrophilic molecules but can also act as a sink to concentrate hydrophobic small molecules). It would be helpful if the Introduction better prepared the reader to make this connection!
Our Response: This is a very helpful suggestion- we neglected to make that connection in the introduction in our original submission. We have added the following sentence in the last section of the introduction, which should tie it all together (new sentence underlined):

….Upon staining animals with a variety of dyes that bind the pharyngeal cuticle, we find that both pgp-14 and sms-5 mutants have a specific deficit of Nile Red staining within the pharyngeal cuticle relative to the wild type. Nile Red fluoresces red only in the presence of polar lipids (Greenspan and Fowler, 1985). We infer that PGP-14 and SMS-5 are necessary for the establishment of a polar lipid barrier within the pharyngeal cuticle.

Our data suggest a model whereby PGP-14 pumps polar lipids into the developing cuticle in a manner that is dependent on a plasma membrane rich in sphingomyelin. We posit that it is this lipid barrier that acts as a sink for the hydrophobic small molecules that form the birefringent crystals within the pharynx cuticle whilst also acting as a barrier for the accumulation of other small molecules into the animal. Transporting phospholipids into the digestive tract of animals by p-glycoproteins may therefore be ancient role that is shared by PGP-14 and ABCB4 alike.

Reviewer #2-Comment 3: As mentioned above, other worm tissues are coated by other types of aECM that also have lipid-based barriers - please state explicitly whether the tested crystal toxins do or do not concentrate in those other locations too. And if not, what is the hypothesis to explain this difference?
Our Response: With respect to the first point of stating explicitly whether the crystals form exclusively in the anterior pharynx, in the third paragraph of the introduction of the original and revised versions of the manuscript, we call attention to the fact that the objects form nowhere else in the animal. We write, *'We have demonstrated that these molecules form birefringent crystals or globular spheres in association with the anterior pharynx and nowhere else in the animal (Kamal et al., 2019).'*

With respect to the reviewer's second point of providing an explanation for this exclusivity, we were reluctant to add additional speculation to the manuscript. But, we understand the rationale for requesting us to do so. Here it is.

We reason that objects only form in the anterior cuticle because of the special combination of a lipid sink, mediated in part by PGP-14, and the amyloid-like properties of the pharynx cuticle itself, which we described in our recent Kamal *et al.*, 2022 eLife paper. We tie this together by revising the second paragraph of the discussion (revised sentence is underlined):

The lipid content within a cuticle can fulfill a barrier role likely because its hydrophobicity prevents the free passage of hydrophilic compounds. Paradoxically, the PGP-14-mediated polar lipid deposition in the anterior pharynx may acts as a sink for the accumulation of hydrophobic small molecules. We speculate that the amyloid-like material within the pharynx cuticle (Kamal et al., 2022) may seed the formation of crystals from the concentrated small molecules, depending

on the small molecules' physicochemical properties. We suspect that crystal formation is unlikely to occur in the wild given that we observe it in the laboratory only at micromolar concentrations. Natural products are unlikely to be common at such high concentrations in the wild. Lipid barriers are therefore unlikely to drive small molecule crystallization in the worm's natural habitat.

Reviewer #2-Comment 4: Figure 4: The labelling of figure panels is fairly confusing here. Non-independent images (e.g. different channels from same image) should not have different panel letters.
Our Response: We apologize for the deficient clarity. We have modified the panel letters and the legend according to the suggestion. We hope this improves clarity.

Reviewer #2-Comment 5: Figure 5: revise title so that it doesn't imply that "genes" are secreted.
Our Response: We have revised the title as follows:

A Temporal Expression Map of 226 Genes whose Protein Products are Predicted to be Secreted and/or Expressed by the Pharynx.

Reviewer #2-Comment 6: lines 207-208: The pgp-14 expression pattern is coincidental with established markers of the pharyngeal epithelium that INCLUDE..."
Our Response: We thank the Reviewer for catching the error and have made the correction.

Reviewer #2-Comment 7: acknowledgements: "David Hall for Zeynep Altun" ?? DH is an author so shouldn't be thanked, not sure what this means.
Our Response: We apologize for this error from an older draft. We have removed the mistaken acknowledgement and thank the Reviewer for catching that.

Reviewer #3-Opening Remarks: This manuscript, entitled "The ABCB4 Homolog PGP-14 Establishes a Lipid Permeability Barrier within the *C. elegans* Pharyngeal Cuticle", fills important gaps in current knowledge about the role of ABC transporters in the nematode *C. elegans*.

These data are original, since *pgp-14* deficiency is associated with resistance to small hydrophobic compounds, that forms crystals usually toxic in parental worms. Whereas the opposite is expected for conventional Pgp subtrate drugs, for which Pgp deficiency is generally associated with drug hypersensitivity, and overexpression with drug resistance.

The authors made several interesting observations, and conclude that *pgp-14* in *C. elegans* is the functional homolog of the mammalian transporter ABCB4. It is particularly intriguing that among the 14 Pgps in *C. elegans*, only *pgp-14* is essential for crystal formation and toxicity when exposed to the small hydrophobic toxic compounds wact. They identified several *pgp-14* null mutants which are resistant to the lethal effects of wact compounds, and there was no crystal formation in response to wact exposure in these mutants, as generally observed in the pharyngeal cuticle of parental worms. Interestingly, similar phenotype was also observed in worms deficient for sphingomyelinase SMS-5.

They provide a number of convincing evidences that PGP-14 may function coincidently with SMS-5 as a regulator of lipid membrane integrity. PGP-14 co-regulates and locates close to SMS-5 in the pharynx cells. PGP-14 deficiency leads to reduction of polar lipid as shown by Nile Red staining, and more specifically *pgp-14(0)* have lower sphingomyelin abondance in the pharynx. In addition, lower penetration of Hoechst 33342 withing the pharynx cuticle of mutant worms is in favor of an altered lipid and Pgp transport function integrity. In agreement, CelPgp-14 protects the worms from several drug toxicity which is in favor of a drug efflux export function.
Our Response: We thank Reviewer #3 for the detailed summary and very kind words.

Reviewer #3-Comment 1: A major question remains: does Pgp-14 act indirectly on drug transport by changing lipid membrane composition? The data showing that Pgp-14 protects from several drug toxicity support a drug transport activity.
Our Response: We have long contemplated the question of whether PGP-14 is a drug transporter that simply exports the crystallizing small molecules from the pharynx epithelium. Believe me, 'PGP-14 as a drug pump' would have been a simpler paper to write because, <u>in the absence of any evidence</u>, the simplest model to account for PGP-14's role in small molecule crystallization is that PGP-14 is pumping the crystalizing molecules from the epithelia that underlies the cuticle where the molecules concentrate and fall out of solution. However, the evidence simply does not allow us to support this model.

In the results section, we describe the tests of the hypothesis that PGP-14 transports the crystalizing molecules (directly or indirectly). We wrote the following:

> If this were true, then we might expect an increase in the abundance of the respective small molecules in tissue of *pgp-14(0)* mutants relative to wild type animals because the crystalizing molecule cannot be pumped out… First, we measured the accumulation of wact-190 by mass-spectrometry in *pgp-14(0)* mutants and the wild type. There is a significant decrease in wact-190 abundance in the *pgp-14(0)* background relative to wild type, and this deficit is rescued by transgenically-expressing PGP-14 in the background of the *pgp-14(0)* mutant (Figure 3F). Second, we asked whether the fluorescence of object-forming wact-40, wact-43, and wact-469 accumulates in the epithelium of *pgp-14(0)* mutants relative to wild type (Figure 3G). We observe no obvious wact-469 accumulation in the tissue surrounding the pharyngeal cuticle in the *pgp-14(0)* mutant background, even when the photos are over-exposed. Third, our chemoinformatic analysis of the crystal-forming compounds (Kamal et al., 2019) shows that only 8% (4/48) are predicted to be ABCB1/MDR1 substrates (using the Swiss-ADME online tool (Daina et al., 2017)). (Supplementary Data File 1). Coincidentally, the fluorescent wact-40 and wact-469 are two of the four crystalizing molecules predicted to be MDR1 substrates, but even these molecules do not accumulate intracellularly in the *pgp-14(0)* mutant (Figure 3G).

Together, these observations argue against the idea that PGP-14 is a drug exporter of wact-190 and other crystalizing molecules.

To further address the reviewer's question, we greatly expand the third paragraph in the discussion to the following (new sentences underlined):

Like all *C. elegans* PGPs, PGP-14 is a member of the ABCB class of whole transporters (Sheps et al., 2004) and is most homologous to human MDR1 (ABCB1) and MDR3 (ABCB4) (see Supplementary Figures 1 and 2). MDR1 is the primary drug efflux pump within human cells (Shen et al., 1986) and can be mutated to confer multi-drug resistance when under negative selection pressure (Juliano and Ling, 1976; Piecuch and Oblak, 2014). An obvious alternative model to explain PGP-14's role in crystal formation is that it pumps crystallizing molecules from the pharynx epithelium, which then precipitate out of solution once concentrated outside of the epithelium. Also in this model, the 188 'hypersensitive' molecules (Figure 7E) are those that are normally pumped out of the pharynx epithelium by PGP-14, but hyperaccumulate in the *pgp-14* mutant.

We do not favour this alternative model for multiple reasons. First, fluorescent molecules that fail to crystalize in the *pgp-14(0)* mutant do not obviously accumulate in the cells that normally express PGP-14 (or any other cells) (see Figure 3G). The same is true for the lack of accumulation of wact-190 in the *pgp-14(0)* mutant relative to the wild type as detected by mass-spectrometry (see Figure 3F). The molecules that crystalize are hydrophobic, and worms cultured in water-based media should act as a sink for their accumulation, which should be readily observable in the *pgp-14(0)* mutant if PGP-14's role is to export these molecules. Second, in *mlt-9(RNAi)* animals, the crystals are tightly associated with the detached pharynx cuticle (see Figure 2T). If the crystallizing molecules were exported from epithelium by PGP-14, the resulting crystals might be randomly distributed within the space created by the detached cuticles of *mlt-9(RNAi)* animals, but that is not what is observed. Third, under this alternative model, the crystallizing molecules might be expected to be more hydrophobic than the 188 hypersensitive molecules reported here. Otherwise, the hypersensitive molecules might also precipitate out of solution once exported from the epithelium. We see no statistical difference in the hydrophobicity between the two groups of molecules (p>0.23; Supplementary Data File 1). Fourth, fewer than 10% of crystal-forming compounds are predicted to be MDR1 substrates. Finally, unlike canonical multi-drug export pumps, there is little evidence to suggest that *pgp-14* expression is xenobiotic-responsive. Despite *pgp-14* being expressed at higher basal levels (along with many other genes) in a strain evolved to resist ivermectin (Menez et al., 2016), large-scale systematic expression analyses show that *pgp-14* expression is not co-regulated with xenobiotic detoxifying enzymes like *pgp-1, -2, -3, -5, -6, -8, -9* and *pgp-13* (Schmauder et al., 2021). Instead, *pgp-14* is co-regulated with genes involved with pharynx function such as *myo-1* and *myo-2* (Schmauder *et al.,* 2021). Consistent with these findings, a second study demonstrated that the upregulation of *pgp-14* expression is independent of a major xenobiotic-responsive nuclear receptor, NHR-8, unlike several other *pgp* genes that are NHR-8-dependant (Guerrero *et al.,* 2021). The upregulation of *pgp-14* in the aforementioned evolved strain may contribute to a fortified lipid barrier or may simply be inconsequential. For some of these points, we can conceive of different explanations that would account for the observations under a drug-pump model, but these explanations are cumbersome and non-parsimonious. For these reasons, we favour the model that PGP-14 contributes to a polar lipid barrier, which parsimoniously accounts for all of the observations.

Reviewer #3-Comment 2: It remains unclear whether this is due to the specificity of tissue expression in the pharyngeal cuticle or to the characteristics of this specific transporter. An additional experiment that would be informative is to rescue Pgp-14 deficient worms with a Pgp other than Pgp-14, chosen from the 13 others in *C. elegans*, and address it to the pharyngeal cells. This could be CelPgp-1 (Jin et al, 2012)... or any other Pgp known to be involved in drug transport. This will answer the question of the specific role of Pgp-14. It may be that all Pgp's can do the job, as long as they are expressed in the appropriate place.

Our Response: Our analyses of all *C. elegans* PGPs shows that PGP-14 is the only PGP that is required for crystal formation (original Supplementary Figure 3). The reviewer, however, is asking a different but interesting question: Is it that PGP-14 is the only PGP capable of facilitating crystal formation, or might other PGPs substitute, if only they were localized to the apical membrane of the pharynx epithelium.

Nearly 20 years ago, David Ballie's group built transcriptional reporters of most of the *pgp* genes in *C. elegans* (see PMID 15522294). In that paper, he showed that *pgp-2* and *pgp-9* are expressed in the pharynx in addition to *pgp-14*. This observation is supported by the modern single cell sequence data of Jay Shendure, Cole Trapnell, Bob Waterston and colleagues (see PMID 28818938) that we have also analyzed in great detail in our recent eLife paper (see PMID 36259463). Also, *pgp-2* and *pgp-9* oscillate in expression over the larval cycle like *pgp-14*, with *pgp-9* peaking in expression close to *pgp-14* (see our eLife paper (PMID 36259463) and the Hendriks *et al* paper (PMID 24440504)). So, one might assume that PGP-2 and PGP-9 clearly cannot substitute for PGP-14. However, the RNA seq data shows that *pgp-14* is expressed 150-fold more than *pgp-2* and 230-fold more than *pgp-9* in the pharynx. As reported in our *pgp-14* manuscript, *pgp-14* is one of the highest expressed genes in the pharynx, and certainly more than any other *pgp* (the next-highest expressed *pgp* is *pgp-2*). So, we can refine the reviewer's question to ask whether any other PGPs can substitute for PGP-14 if it were not only expressed in the same tissue and at the same time, but also at the same level.

In humans, the PGP ABCB1 (aka MDR1, aka PGP) is a primary drug exporter, but can also export polar lipids at a lower efficiency (see PMIDs 8898203, 11389609, 12071854). Conversely, the PGP ABCB4 (aka MDR3) exports polar lipids, but can also export small molecule drugs at a lower efficiency (see PMIDs 10918072, 25133187). Presumably, if expressed at a high enough level in the right place and at the right time, one could substitute for the other. This would show that, for example, ABCB4 <u>can</u> act as a multi-drug transporter, but it would not show whether it normally does so.

We are making the same argument here. Showing that *C. elegans* PGP-X can rescue *pgp-14(0)*'s inability to form crystals will not tell us anything new about PGP-14. Even if PGP-X is characterized to be a drug exporter, at high enough levels, PGP-X might exhibit other functions.

To address the reviewer's point, we have included this new sentence in relevant results section in the revised manuscript:

> *We cannot exclude the possibility that if expressed at the correct time in the correct tissues and at high enough levels, that other* C. elegans *PGPs might be able to substitute for PGP-14.*

Reviewer #3-Comment 3: The proposed model in the discussion seems to contradict the assertion that Pgp-14 is the functional homolog of ABCB4. While ABCB4 unambiguously transports PL, the authors propose that Pgp-14 does not. Rather SMS-5 produces PL in the lipid membrane, and the polar lipid enrichment enhances the activity of Pgp14. If this is the case, Pgp-14 will not be the exact functional homologue of ABCB4. Regarding the sequence comparisons, it is difficult to conclude of a sequence homology specific of PGP-14 and ABCB4. I will rephrase the title to moderate this aspect.

Our Response: After further reflection on the reviewers' comments, we have changed the title from: The ABCB4 Homologue PGP-14 Establishes a Lipid Permeability Barrier within the *C. elegans* Pharyngeal Cuticle, to the simpler, 'PGP-14 Establishes a Polar Lipid Permeability Barrier within the *C. elegans* Pharyngeal Cuticle'.

We have also changed the following sentence from the abstract.

Original: Here, we show that <u>the ABCB4 homolog</u> PGP-14 functions coincidently with the sphingomyelin synthase SMS-5 to establish a xenobiotic barrier within the pharyngeal cuticle of the nematode *C. elegans*.

Revision: Here, we show that <u>the predicted p-glycoprotein</u> PGP-14 functions coincidently with the sphingomyelin synthase SMS-5 to establish a xenobiotic barrier within the pharyngeal cuticle of the nematode *C. elegans*.

Nowhere else do we explicitly state that PGP-14 is likely to be orthologous to ABCB4. We simply highlight similarities between the two proteins and let the reader draw their own conclusions.

Reviewer #3-Comment 4a: SFig1, comparing with other Pgp, the residues involved in PL binding on ABCB4 are not obviously present in Pgp-14. 1- The primary sequence of the protein does not allow necessarily to align hotspot residues. Alphafold 3D model structure would be more accurate.
Our Response: We have added the requested comparison in Supplementary Figure 1c and 1d. The structural alignment confirms the primary sequence alignment.

Reviewer #3-Comment 4b: 2- This does not exclude the possible transport of other PL species. What do we know about PL composition in *C. elegans*? Is there a specific PL profile composition and species when compared to mammals?
Our Response: With respect to sphingomyelin, nematodes (including *C. elegans*) have a clear difference when compared to other animals including humans. Nematodes produce iso-branched sphingoid bases (i.e., branched sphingomyelin), which is distinct from the unbranched form harboured by other animals (see PMID 30155209).

With respect to phosphatidylcholines, there is no evidence that there are any structural differences between nematodes and other animals. Any differences may simply be in relative abundance of the different phosphatidylcholine species, and differences are likely especially evident when different cell types are compared, both within and between species. For example, Satouchi et al, 1993 (PMID 8231660) showed that bulk *C. elegans* have phosphatidylcholine alkyl chains that are predominantly (i.e., >92%) 18:0 and 19:1. By contrast, van Berge Henegouwen et al., 1987 (PMID 3111757) found only phosphatidylcholine alkyl chains within human bile to be of 18:0 (5.5%) and none of 19:1 were reported.

Without a systematic, modern direct comparison, which is beyond the scope of this manuscript, this type of question is difficult to address thoroughly, and even if we could, it would be at the level of the whole animal and not specific to the pharynx epithelium, or more importantly, the pharynx cuticle itself.

We remind the reviewer of the following statement in the discussion, which has been revised to add additional caution (revisions are underlined):

> *While our NT-lysenin results suggest that PGP-14 may flop PCs to the outer leaflet along the pharynx plasma membrane (see Figure 6J), it is entirely possible that PGP-14 exports <u>other</u> phospholipids, <u>which may or may not include</u> PCs, to the developing pharynx cuticle.*

Reviewer #3-Comment 5: Discussion line 385-386: the authors exclude the possibility that Pgp-14 transports wact crystals. In another way, Pgp14 may be able to export the free wact compounds that accumulate under the cuticle in wild type animals and form crystal that causes the toxicity. In Pgp14(0) the compound is not exported and may diffuse in the animal in a non-toxic form. Can you discuss this point?
Our Response: We go into detail on this point in Reviewer #3's Comment 1 above.

Reviewer #3-Comment 6: It would be of interest to discuss some of the published work reporting CelPgp14 expression and/or drug toxicity (Janssen et al, 2012; Guerrero et al 2021; Menez et al, 2016).
Our Response: We have added the following to the relevant section of the discussion:

> *Finally, unlike canonical multi-drug export pumps, there is little evidence to suggest that pgp-14 expression is xenobiotic-responsive. Despite pgp-14 being expressed at higher basal levels (along with many other genes) in a strain evolved to resist ivermectin (Menez et al., 2016), large-scale systematic expression analyses show that pgp-14 expression is not co-regulated with xenobiotic detoxifying enzymes like pgp-1, -2, -3, -5, -6, -8, -9 and pgp-13 (Schmauder et al., 2021). Instead, pgp-14 is co-regulated with genes involved with pharynx function such as myo-1 and myo-2 (Schmauder et al., 2021). Consistent with this finding, a second study demonstrated that unlike several other pgp genes, the upregulation of pgp-14 expression is independent of a major xenobiotic-responsive nuclear*

receptor, NHR-8 (Guerrero et al., 2021). The upregulation of pgp-14 in the aforementioned evolved strain may contribute to a fortified lipid barrier or may simply be inconsequential.

Reviewer #3-Comment 7: It is well known that mRNA levels do not necessarily correlate with protein levels. I would recommend tempering this aspect in the manuscript.
Our Response: In response to this comment, we have added and modified the following sentences in the results section (additions are underlined):

> <u>In general, there is a reasonable but imperfect correlation between mRNA expression levels and protein abundance in *C. elegans* (R_S of +0.59 (Schrimpf et al., 2009)).</u> <u>Regardless, the</u> peak in *<u>pgp-14</u>* mRNA expression is consistent with the increase in abundance of tagged-PGP-14 protein during the molt (Figures 4G and 4H; Supplementary Figure 5).

Reviewer #3-Comment 8: According to the table 4 in sup data file showing which wact- are Pgp substrates according to the Swiss ADME, would it be a rationale emerging from the wact compound structure that may explain their Pgp substrate specificity?
Our Response: Any molecule that is predicted to be a substrate of MDR1 by SwissADME has a combination of physicochemical features that, by definition, must be distinct from those that are not predicted to be a substrate. The MDR1 substrate predictor is described in the SwissADME publication (PMID 28256516) and is referred to in the manuscript.

As described in our response to Reviewer #3's Comment 1, there is no evidence that the crystalizing wactives are PGP-14 substrates, so it is difficult to address the reviewer's comment above. As stated in the manuscript: *'Coincidentally, the fluorescent wact-40 and wact-469 are two of the four crystalizing molecules predicted to be MDR1 substrates, but even these molecules do not accumulate intracellularly in the* pgp-14(0) *mutant (Figure 3G).'*

Reviewer #3-Comment 9: Fig3F shows the mass-spectrometry analyses which seem to correlate with the crystal content. Can you provide evidence that mass-spectrometry can measure free wact compounds and provide the yield of the extraction, and the limit of detection/quantification of the technique.
Our Response: The extraction efficiency of wact-190 was 89% in MTBE. Our method was validated using external calibration curve with an internal standard of triamcinolone acetonide. The assay was linear between 10 uM and 50 uM (exhibiting non-linearity at 100uM). We estimated the LOD from the equation LOD= 3.3*Sy/S. By this measure the assay would have an LOD of 5 uM. We have included this information in the revised methods section.

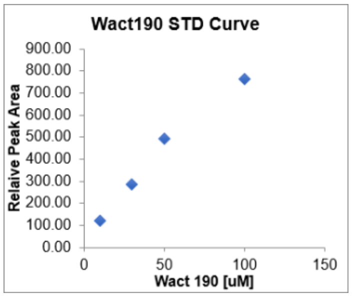

Reviewer #3-Comment 10: It is unclear why in some experiments wact-190 is used and in others, wact-469.
Our Response: We address this comment by adding the following sentences in the relevant results section:

> *The workhorse molecule that we use for most of our crystal studies is the non-fluorescent wact-190 because of its long-term stability. However, for some experiments, including this*

pulse chase experiment, we use wact-469 because its fluorescent properties make it easier to track.

Reviewer #3-Comment 11: Fig 3G represents fluorescence of wact compound. What is the difference when compared with the location of crystals showed by birefringence in fig3C
Our Response: Birefringence is the redirection of polarized light that is visible through a second polarized filter. So, if there is a collection of crystals (of any type) randomly thrown on a slide, only the fraction of the crystals that are aligned with the two filters in such a way that the redirected polarized light can escape the second polarized filter will be visible through the second filter. By contrast, all fluorescent crystals will be visible when observed with the appropriate fluorescent filters. In other words, only a fraction of the crystals that are visible with fluorescence will also be visible with birefringence. A great example of this is with wact-469 in Figure 1b of our Kamal et al., 2019 paper.

Reviewer #3-Comment 12: Title of Figure 5. Change “secreted” by “expressed “.
Our Response: We have changed the title to *‘A Temporal Expression Map of Genes whose Protein Products are Predicted to be Secreted and/or Expressed by the Pharynx.’* We felt it necessary to keep ‘Secreted’ in the title because the map focuses on genes whose respective protein products are predicted to be secreted.

Reviewer #3-Comment 13: SFig1B: Please indicate which Pgp region is shown in this panel B
Our Response: For additional clarity, we have added the residue number of the last PGP-14 residue shown to the right of each alignment in the revision. In the revised legend, we write*, ‘In each alignment, the last residue of PGP-14 is indicated to the right for reference.’*

Reviewer #3-Comment 14a: SFig2. The phylogenetic Tree Analysis of *C. elegans* and Human ABCB. Precise on which aa sequence the pairwise blast identity comparisons was performed.
Our Response: To add clarity, we have revised the phrasing of the relevant original sentence in the legend to the following (additions are underlined): *‘The Uniprot entries used to create the phylogenetic tree and to make the pairwise blastp comparisons are the same described in Supplementary Figure 1.’*

Reviewer #3-Comment 14b: SFig2. Indicate what the % corresponds to: identity or similarity.
Our Response: To add clarity, we have revised the phrasing of the relevant original sentence in the legend to the following (additions are underlined): ‘*The pairwise blastp percentage identity ~~comparisons~~ (using NCBI protein blast tool) between PGP-14 and the four human ABCB family members is ~~are~~ indicated next to the respective human protein names.’*

Reviewer #3-Comment 15: Line 405: The gene products are RNA and not proteins: this should be clarified since transcript levels do not always reflect protein levels.
Our Response: We have changed ‘gene products’ from the original line 405 to ‘mRNA’.

Reviewer #1-Opening Remarks I: The manuscript 'The ABCB4 Homolog PGP-14 Establishes a Lipid Permeability Barrier within the *C. elegans* Pharyngeal Cuticle' by Kamal et al is an extension of their previous work (Kamal et al. 2019). In their previous work, they had shown that worm-active compounds form crystals or spheres and some of them cause lethality. Compounds such as wact-190 damage the plasma membrane. The previous study also reported a forward genetic screen to find genes that prevent lethality caused by wact. They had earlier identified mutations in sms-5 and pgp-14 to be involved in protection from crystals. This specific study is a further characterization of *pgp-14* allele and complementation to show that the ABCB transporter is also protective. Pgp-14 is expressed in the same tissues and at the same time (in the context of molting) as sms-5. They work in the same pathway in protecting worms from toxic worm-active compounds. This study lacks originality, it is a description of pgp-14 which works in the same pathway as sms-5 previously described. In fact, we are left in the same place where the previous study (Kamal et al. 2019) ended.

Our Response: We are sorry that Reviewer #1 does not share Reviewers 2 and 3's enthusiasm for the work, who wrote:

> 'This manuscript…fills important gaps in current knowledge about the role of ABC transporters in the nematode *C. elegans*.', and,
>
> 'These data are original, since *pgp-14* deficiency is associated with resistance to small hydrophobic compounds, that forms crystals usually toxic in parental worms.', and,
>
> 'Here Kamal et al provide convincing evidence that the ABCB4-type transporter PGP-14 is needed to establish a proper xenobiotic barrier in the *C. elegans* pharynx cuticle, and that it acts in a common process with the sphingomyelin synthase SMS-5.', and,
>
> 'Overall, the data appear rigorous and represent a significant step forward in understanding the biology of the pharyngeal cuticle - it will be of broad interest to cell and matrix biologists, especially those interested in lipid transporters.'

The formation of small molecule crystals in the context of the worm is a complex process. Understanding the role of the gene products that facilitate crystal formation is an equal challenge. Yet, our manuscript makes headway in understanding of both of these issues. In the hopes of tilting the Reviewer #1's perspective, we summarize some of the key findings reported in this manuscript that i) extend our understanding of PGP-14 and crystal formation, and ii) illustrate how our work makes original contributions:

<u>2019 status of knowledge on the location of crystal formation:</u> We speculated that the crystals form in the context of the plasma membrane of the marginal cells.

<u>Novel insight on the location of crystal formation present in Kamal *et al* 2023</u>: We provide three independent lines of evidence that crystals form in association with the pharynx cuticle, and not the underlying epithelium.

(i) Figures 2A-2K show TEM images that reveal the crystals to be associated with the cuticle and that their extension deforms the underlying epithelium. *This finding is entirely original.*
(ii) Figures 2L-2R show a pulse-chase technique that reveals that crystals are shed in molting animals, but not in non-molting adults. *We consider this to be a novel result and clever experiment that contributes to the originality of the story.*
(iii) Figures 2S and 2T show that crystals exist in the partially shed cuticle of *mlt-9(RNAi)*-treated animals. *We think this to be a novel result and inventive exploitation of Gary Ruvkun's mlt-9 discovery and contributes to the originality of the paper.*

<u>2019 status of knowledge on *pgp-14* alleles:</u> In describing our screen for genetic suppressors of crystal formation, we mentioned, but did not describe in any way, the isolation of *pgp-14* alleles in the spirit of transparency. What we wrote was, '*Sequencing the genomes of the 46 mutants revealed 29 alleles of pgp-14, whose contribution to small molecule accumulation will be discussed elsewhere.*'

<u>Status of knowledge on *pgp-14* alleles present in Kamal *et al* 2023:</u> In the present manuscript, we do exactly what we said we would do- describe the details of those 29 alleles. In fact, our collection of *pgp-14* alleles (Figure 3a and 3b etc) is the largest of any collection of functionally consequential alleles reported for

any Pgp in *C. elegans*. *This collection is highly original and a valuable resource for the Pgp community (not just for those interested in C. elegans Pgps).*

<u>2019 status of knowledge on *pgp-14* expression:</u> Before our work, knowledge of *pgp-14* expression came from one low resolution image presented 19 years ago in David Baillie's foundational paper on Pgps (see PMID 15522294).

<u>Novel insight on *pgp-14's* expression pattern presented in Kamal *et al* 2023:</u> We describe *pgp-14*'s expression pattern in vastly more detail than what has been done previously.

(i) We have generated a functional fluorescently tagged transgene of PGP-14 and show that it localizes to the apical plasma membrane of the cells that generate the pharyngeal cuticle.

(ii) We have extracted and processed single cell sequence data (from Cao *et al.* 2017 that provided expression data on all *C. elegans* genes) in a novel manner (see Figure 4A-E) that supports the conclusions drawn from our PGP-14 reporter (and demonstrates overlap with the *sms-5* expression pattern).

(iii) *We present a novel approach* to analyzing the similarity of the *pgp-14* expression pattern to all other genes in the genome, and show that that *pgp-14*'s expression pattern overlaps highly with that of known markers of the pharynx epithelial cells and *sms-5* (see Figure 4F).

(iv) We exploit the data from the spatiotemporal map that we published in eLife (Kamal *et al*., 2022) to show how closely *pgp-14* and *sms-5* are expressed temporally. *This spatiotemporal correlation is not self-evident and is an original finding.*

<u>2019 status of knowledge on *pgp-14* and *sms-5*'s contribution to crystal formation:</u> In 2019, we had no idea how *sms-5* was contributing to crystal formation. We speculated about how a sphingomyelin-rich plasma membrane might be more suitable to absorb and concentrate the small molecules that go on to form crystals. Our present analysis clearly shows that the crystals don't even form in the plasma membrane, so our 2019 interpretation of how *sms-5* contributes to crystal formation is in need of updating to reflect where crystals form, which the present manuscript provides.

<u>Status of knowledge on *pgp-14* and *sms-5*'s contribution to crystal formation in Kamal *et al* 2023:</u> We show in the present manuscript for the first time that *pgp-14* and *sms-5* are required for the accumulation of Nile red dye, which fluoresces red only in the presence of polar lipids (PMID 4031658). The *pgp-14* and *sms-5* mutants behave this way only with respect to Nile red and not other dyes like calcofluor white, Congo red (and several others that we have looked at but not presented). For this reason, and other pieces of data presented in the manuscript, we surmise that the two gene products are required for the deposition of polar lipids into the cuticle. *This is an entirely original result*.

We hope that highlighting these points might help the reviewer understand why we feel that this manuscript does indeed present original work and furthers our understanding of both small molecule crystal formation and *pgp-14* and *sms-5*'s role in facilitating the phenomenon.

Reviewer #1-Opening Remarks II: The fact that lipid layer of the cuticle (as well as collagens) serve as a permeability barrier for genotoxic compounds has been demonstrated by various labs.
Our Response: As far as we know, no one has done a molecular genetic characterization of the structure of the chitin-based pharynx cuticle in any nematode beyond our group. Hence, additional information on this structure adds value to our foundational knowledge.

Reviewer #1-Opening Remarks III: Components of the lipid layer of the cuticle, sphingomyelin synthases and glycoproteins such as *pgp-14*, have long been ascribed to this function. In absence of findings that take the field forward, it is difficult to be enthusiastic about this study.

Our Response: While we appreciate diverse points of view, our view is that the novel contributions that we have made to the understanding of small molecule crystal formation and *pgp-14* function, as highlighted in detail above, meets or exceeds the bar that is set by papers that are routinely published in PLoS Genetics.

Reviewer #1-Comment 1a: Sms-5 was shown to have less sphingomyelin in their previous study.
Our Response: The Reviewer is correct, and we draw attention to this point in the original manuscript, where we write:

> *If PGP-14 flop PCs to the outer leaflet, then pgp-14(0) might also have a deficit in SM abundance. Indeed, we observe a significant decrease in GFP::NT-lysenin signal in the pgp-14(0) mutant background (p=0.004; Fig 6I-6J). The fold decrease in clustered SM in pgp-14(0) is not as dramatic as that observed in the sms-5 null background (Kamal et al., 2019). These observations suggest that SMS-5 uses other substrates beyond what PGP-14 may provide, which is not unexpected. Regardless, this data is consistent with the model that PGP-14 flops PC in vivo.*

Reviewer #1-Comment 1b: A similar phenotype has been shown for *pgp-14* in a single worm image.
Our Response: We apologize in advance if we are misinterpreting this statement, but we present data for the clustered sphingomyelin experiment beyond a single worm image (Figure 6I). In the neighboring Figure 6J, we present the quantification of 194 wild type and 230 mutant animals for the relevant clustered sphingomyelin data. The style of showing a representative set of images alongside detailed data is typical of display items that we are familiar with.

Reviewer #1-Comment 1c: There is no quantification or detergent sensitivity assay.
Our Response: With respect to experiments related to detergent sensitivity, we presented none in this manuscript. As the Reviewer correctly notes with respect to the NT-Lysenin (clustered sphingomyelin) experiment, the difference between *pgp-14(0)* and wild type is less than the difference between *sms-5(0)* and wild type (from Kamal et al., 2019). We therefore expected that *pgp-14(0)* would exhibit a much smaller hypersensitivity to detergents than what we previously found for *sms-5(0)*, which would be accounted for by the statement highlighted above, '*These observations suggest that SMS-5 uses other substrates beyond what PGP-14 may provide, which is not unexpected.*'

Reviewer #1-Comment 2: Does supplementation of sphingomyelin in the plates affect drug sensitivity or crystal formation. Does supplementation of PC affect crystal formation? Is it dependent on both sms-5 and pgp-14?
Our Response: We wanted to try this experiment with sphingomyelin for our 2019 Nature Communications paper on SMS-5 but there were two problems. First, nematode sphingomyelin is structurally distinct from vertebrate sphingomyelin in that nematodes produce iso-branched sphingoid bases (i.e., branched sphingomyelin), in contrast to the unbranched form generated by vertebrates (see PMID 30155209). Of course, nematode sphingomyelin is not commercially available, leaving us only with vertebrate options. Second, we could not get the sphingomyelin into an aqueous solution to any significant concentration. Not surprisingly then, we failed to see rescue with the exogenous addition of vertebrate sphingomyelin to the *sms-5(0) C. elegans* mutants.

In response to the reviewer's question here, we returned to this type of experiment with phosphatidylcholine (PC). Here, we used Soy PC that has a distribution of fatty acids as follows: 16:0 (14.9%), 18:0 (3.7%), 18:1 (11.4%), 18:2 (63.0%), and 18:3 (5.7%). Worm PCs are not obviously structurally different than vertebrate PCs (or the PCs in this mixture)*, but the chain lengths that might be relevant to our experiments are of course unknown. To try and overcome solubility issues, we used a protocol to make PC

micelles used by a colleague in our institute (Chris Yip- see PMID 20197035) and under his lab's guidance. We tested the ability of these micelles to rescue the crystal-forming deficit of the *pgp-14(0)* mutant (and wt controls) under a number of different concentrations, but we saw wildly varying results. In some trials the PC micelles would indeed rescue the *pgp-14(0)* phenotype, and in others, the PC micelles failed to rescue and even suppressed crystal formation in wild type. One interpretation of this latter result is that the PC micelles were absorbing the hydrophobic wact-190-crytal forming compound. In the end, this avenue was fruitless.

* For example, Satouchi et al, 1993 (PMID 8231660) showed that bulk *C. elegans* have phosphatidylcholine alkyl chains that are predominantly (i.e., >92%) 18:0 and 19:1. By contrast, van Berge Henegouwen et al., 1987 (PMID 3111757) found only phosphatidylcholine alkyl chains within human bile to be of 18:0 (5.5%) and none of 19:1 were reported.

Reviewer #1-Comment 3: The evidence that PGP-14 flops sphingolipid from one lipid layer to another is not provided. Indeed, the probe used, GFP_NT-lysenin does not report flopping at all, it just indicates binding to sphingolipids.

Our Response: The logic of our argument is as follows. GFP::NT-lysenin is a protein probe that binds to clustered sphingomyelin (Ishitsuka *et al.,* 2004; Yilmaz *et al.,* 2018). We used the GFP::NT-Lysenin probe <u>without permeabilizing cells</u>. Hence, any GFP::NT-lysenin signal should come from the surface of cells. We observe a decrease in GFP::NT-lysenin in the *pgp-14(0)* mutant relative to the wild type. Given that the active site of sphingomyelin synthases is extracellular (Tafesse et al., 2006), one interpretation of the decreased GFP::NT-lysenin signal that we see in *pgp-14(0)* mutants is that PGP-14 normally provides SMS-5 with one of its substrates, phosphatidylcholine. We have modified the relevant section to add clarity and additional cautiousness (revisions are underlined):

> *Given the homology between PGP-14 and ABCB4, we asked whether PGP-14 might flop PCs in vivo. We previously demonstrated that sms-5(0) has a deficit of clustered sphingomyelin (SM) on the surface of the pharynx epithelium (Kamal et al., 2019) using a protein probe called GFP::NT-lysenin (Ishitsuka et al., 2004; Yilmaz et al., 2018) <u>on animals whose cells were not permeabilized</u>. The active site of the SM synthases resides on the outer leaflet of membranes where it uses PC as a substrate to make SM (Tafesse et al., 2006). If PGP-14 flop PCs to the outer leaflet, then pgp-14(0) might also have a deficit in SM abundance <u>as detected by a decreased GFP::NT-lysenin signal</u>. Indeed, we observe a significant decrease in GFP::NT-lysenin signal in the pgp-14(0) mutant background (p=0.004; Fig 6I-6J). The fold decrease in clustered SM in pgp-14(0) is not as dramatic as that observed in the sms-5 null background (Kamal et al., 2019). These observations suggest that SMS-5 uses other substrates beyond what PGP-14 may provide, which is not unexpected. Regardless, this data is consistent with <u>the hypothesis that</u> PGP-14 <u>may</u> flop PC in vivo.*
